# Comparative incidence of adverse drug reaction during the first and subsequent year of antiretroviral therapy in a Nigerian HIV infected Cohort

**DOI:** 10.4314/ahs.v21i3.10

**Published:** 2021-09

**Authors:** Isaac O Abah, Wetkos D Dayom, Dauda A Dangiwa, Roseline Aderemi-Williams, Joseph Anejo-Okopi, Oche O Agbaji, Phyllis Kanki, John C Aguiyi

**Affiliations:** 1 Department of Clinical Pharmacy and Pharmacy Practice, Jos University Teaching Hospital/University of Jos, Jos, Nigeria; 2 Department of Clinical Pharmacy & Biopharmacy, Faculty of Pharmacy, University of Lagos, Idiaraba Campus, Lagos, Nigeria; 3 Department of Microbiology, University of Jos, Jos, Nigeria; 4 Department of Medicine, University of Jos/Jos University Teaching Hospital, Jos, Nigeria; 5 Department of Immunology & Infectious Diseases, Harvard T. H. Chan School of Public Health, Boston, MA, USA; 6 African Centre of Excellence for Phytomedicine Research and Development University of Jos, Jos, Nigeria

**Keywords:** Adverse drug events, antiretroviral therapy, drug toxicity, sub Saharan Africa

## Abstract

**Background:**

Despite close to two decades of antiretroviral therapy (ART) in Nigeria, data on late on-onset ART-associated adverse drug reactions (ADRs) are sparse.

**Objectives:**

To describe early and late-onset ADRs and compare their incidence in an outpatient HIV positive Cohort on ART.

**Method:**

We described the incidence of clinical ADRs identified and documented in an outpatient clinic cohort of HIV-positive patients treated between June 2004 and December 2015 at a tertiary health facility in Nigeria. Incidence rates of ADRs during the first and subsequent years of ART were compared.

**Results:**

of the 13,983 patients' data analyzed, 9317 were females (66%), and those in the age bracket of 25 to 45 years made up 78% of the studied population. During 52,411 person-years (py) of ART, 1485 incident ADRs were recorded; Incidence rate (IR) 28.3 (95% confidence interval [CI] 26.9:29.8) ADRs per 1000 person-years (py) of ART. The IR of ADRs was about two times higher in the first year of ART compared to subsequent years of treatment; crude incidence rate ratio (IRR) 1.77 (95% CI 1.59:1.97). Anemia, hypersensitivity reactions, and nervous system disorders had 7, 23, and 5 times higher incidence, respectively, in the first year of therapy, compared to subsequent years.

**Conclusion:**

The first year of ART is the period of highest risk of ADRs. Individual and programmatic treatment success in resource-limited settings requires strategies for early identification and management of ADR during the period of greatest risk of ADRs.

## Introduction

Available data indicate that there is an upsurge in the number of human immune deficiency virus (HIV) infected persons accessing life-saving antiretroviral therapy (ART) globally. As of December 2018, of the 37.9 million people living with HIV worldwide, 23.3 million (61%) were on treatment, more than three times as many as in 2010 1. In Nigerian, roughly one million of the estimated 1.9 million persons living with HIV were on ART in 1500 health facilities across the country 2.

As more patients are commenced on lifelong ART and live longer with HIV, none AIDS-related morbidities such as adverse drug reactions (ADRs) are emerging concerns 3,4. Adverse drug reactions to ART are common, with reported prevalence of 30% to 90%^5^–^8^. In Nigeria, prior studies reported ADR incidences of between 4.6% to 10.4% among individuals receiving ART^9^–^11^. The reported incidence in Nigeria is lower than those reported in other settings, probably due to poor pharmacovigilance practices 11. Antiretroviral therapy-associated ADRs are risk factors for poor health outcomes and mortality in people living with HIV 6,12. Therefore, understanding the pattern of ART-associated ADR will aid their timely identification and management, which could result in improved treatment outcomes.

Antiretroviral therapy-associated ADRs can occur at any time during ART, with some being early in onset, while others are late in onset 13,14. Previous studies of ADRs in Nigeria 9–11 focused more on early-onset ADRs due to a lack of longitudinal data. This study described early and late-onset ADRs in an outpatient clinic cohort of people living with HIV on ART. We compared the incidence of ADRs during the first and subsequent years of treatment. We also modeled the hazard of developing any ART-associated ADR during treatment. This data will be useful in improving the detection of ADRs and focus interventions on the periods of the most considerable risk.

## Method

### Study design

The study was a retrospective cohort study of prospectively identified clinical ADR that occurred among patients on ART at the study site. Analysis of patients' clinical data was conducted from the time of ART initiation (baseline) through the last pharmacy ARV dispense date or end of study period defined as December 2015.

### Study setting and population

The study was conducted at the AIDS Prevention Initiative in Nigeria (APIN) supported HIV treatment center of Jos University Teaching (JUTH) in North Central Nigeria. The outpatient clinic has over a decade of experience in the provision of comprehensive HIV care, treatment, and support services for the city of Jos, and the North Central region of Nigeria. As of December 2015, over 20,000 HIV-infected patients had been cumulatively enrolled in care at the treatment center, and 16,000 had received ART.

HIV-infected patients ≥15 years of age who received treatment at the treatment site between January 2004 and December 2015, and treatment naïve at ART initiation were included in the study. Those excluded from the study included pregnant women who received short-course ART for the prevention of mother to child transmission of HIV and those who picked up antiretroviral medicines only once from the pharmacy. Additionally, patients with insufficient baseline data such as missing first ART dispense date were excluded from the analysis.

Standard of care and adverse drug reaction assessment Eligibility for ART at the treatment facility was based on the treatment guidelines in use at the time of enrollment 15,16. The Nigerian National ART guideline for adults and adolescents is usually aligned with the World Health Organization (WHO) guidelines at the time of patient enrollment^17^,^18^. Eligible patients typically commenced ART that included a non-nucleoside reverse transcriptase inhibitor (NNRTI) backbone of either nevirapine (NVP) or efavirenz (EFV), in combination with two nucleoside reverse transcriptase inhibitors (NRTIs), usually lamivudine (3TC) in combination with zidovudine (AZT), stavudine (d4T), or tenofovir (TDF). Abacavir (ABC) or didanosine (ddI) were also used in select cases. Some patients on TDF received emtricitabine (FTC) in place of 3TC, depending on the formulation available to the program at the time of dispensing. In limited cases, patients initiated ART with a triple NRTI regimen. Prescription records were maintained in an electronic pharmacy database developed for use in the Harvard School of Public Health and AIDS Prevention Initiative (HSPH/APIN) program in Nigeria^19^. Routine clinic visits for clinical review and drug pick-up occurred monthly in the first year of ART initiation and, subsequently, every other month. Patients with complaints had unscheduled visits as necessary. The standard clinical evaluation included a thorough review of symptoms, in which the absence of symptoms or a description of symptoms present was documented on standardized visit forms. If clinical or laboratory toxicity was identified, clinicians completed a program-specific toxicity form, which included detailed information about the suspected medication-related toxicity, date of onset, management, and outcome. The toxicity form contains an extensive checklist of symptoms categorized by body system. ADRs were classified as mild to life-threatening on a four-point scale according to the WHO severity grading 20. Grade one was classified as “mild” and with no limitation of daily activities, grade two as “moderate” with mild to moderate limitation of activities, grade three as “severe” with marked limitation of activities, and grade four as “life-threatening” with extreme limitation of activities and significant medical intervention 20. Furthermore, ADRs were classified as early-onset and late-onset if they occurred within the first year and after one year of ART, respectively. Completed toxicity forms were transferred into the electronic database maintained at clinic to document all clinical, pharmacy and laboratory patient data 19.

CD4+ lymphocyte count was measured by flow cytometry (Partec GmbH, Munster Germany), and HBsAg was determined using Enzyme Immunoassay (EIA) (MonolisaHBsAg Ultra3; Bio-Rad, Hercules, USA). Patients were assigned as being TB positive if it was noted as such in the clinical record.

### Ethical issues

The Jos University Teaching Hospital Institutional review board approved the research protocol, while the Harvard School of Public Health and APIN Public Health Initiative approved the use of secondary data (Ref: OHRP IRB# IRB00011406 dated September 15, 2018). Only patients who had documented consentor the use of their clinical data for research were included in this analysis.

### Statistical analysis

We described frequencies and proportions for categorical variables, while median with interquartile range (IQR) was computed for numerical variables based on results of initial exploratory analysis. The incident rate of ADRs was determined by dividing the number of incident events by the total observation time contributed by patients. Each patient contributed observation time from the start date of ART to the date of the first occurrence of an ADR or date of last ARV pick up or the end of the observation period, defined as the end of December 2015. For incident rates of specific ADRs type, censoring was done at the first report of the ADR type. The incidence rate of ADRs during the first year of ART was compared to the incidence rate during subsequent years of therapy to determine the incident rate ratio (IRR) and 95% confidence interval (CI) for the different ADRs. Cox proportional hazards model was used to predict the hazard of ADR. Baseline characteristics that were significantly associated with ADR in the bivariate analysis (p <0.25), as well as those that were clinically relevant or had a biological plausibility to affect ADRs, were included in a multivariable Cox proportional hazards model. Findings from the model were reported as hazard ratios (HR) and 95% confidence interval (CI) of HR; p-value <0.05 was considered statistically significant. For the multivariate analysis, WHO clinical stage and CD4+ cell count were stratified based on the result of the bivariate analysis. Stata version 13 (College Station, TX) was used for statistical analysis.

## Results

### Participants' characteristics

Between January 2004 and December 2015, 16,012 patients commenced ART at the study site, of which data of 13,983 (85%) patients who met the inclusion criteria were analyzed. The baseline characteristics of the Cohort summarized in Table 1 indicate that majority of the study population were females (66%) and in the age bracket of 25 to 45 years (78%). The dominant HIV transmission risk was heterosexual sex. At the time of ART initiation, approximately one-third of patients had WHO clinical stage three or four disease, while the median (interquartile range [IQR]) CD4 cell count was 153 (78–251) cell/mm^3^. A higher proportion of patients commenced ART with zidovudine+lamivudine+ nevirapine (39%) regimen followed by tenofovir+ lamivudine+nevirapine (n=2665, [19%]), and tenofovir+lamivudine+efavirenz (12%) (Table 2). Less than 10% of the patients commenced ART with abacavir or didanosine containing regimen.

**Table 1:** Baseline demographic and clinical characteristics of study patients (n=13,983)

Characteristics	Sub-group	Frequency	Percent
Sex	Female	9317	66.6
	Male	4666	33.4
Age, years	15–24	771	5.51
	25–45	10865	77.70
	>45	2292	16.39
	Missing data	55	0.39
	Median (IQR)	35 (30–41)	
HIV Transmission risk	Heterosexual	13037	93.21
	Heterosexual/Transfusion	418	3.00
	Transfusion	35	0.25
	MSM	5	0.04
	Heterosexual/ MSM	4	0.03
	Heterosexual/IVDU/Transfusion	1	0.01
	IVDU	1	0.01
	IVDU/Heterosexual	1	0.01
	Unknown	482	3.40
Tuberculosis status	Negative	2070	14.8
	Positive	882	6.3
	Missing data	11031	78.9
HBV status	Negative	9832	70.3
	Positive	2502	17.9
	Missing data	1649	11.8
WHO disease stage	1	4719	33.7
	2	4161	29.8
	3	3483	24.9
	4	818	5.8
	Missing data	802	5.7
CD4 cell count, cells/mm^3^	≤100	4569	32.7
	101–200	4337	31.0
	201–350	3434	24.6
	>350	1579	11.3
	missing data	64	.5
	Median (IQR)	153 (78–251)	

IQR; interquartile range, MSM; men who have sex with men, IVDU; intravenous drug use, HBV; hepatitis B virus, WHO; World Health Organization, CD4; cluster of differentiation 4,

**Table 2:** Antiretroviral regimen used for treatment initiation at Jos University Teaching Hospital

Antiretroviral regime	Frequency	Percent
ABC-AZT-NVP	24	0.2
ABC-3TC-NVP	270	1.9
ABC-3TC-EFV	29	0.2
AZT-ddI-NVP	110	0.8
AZT-3TC-NVP	5434	38.9
AZT-3TC-EFV	1035	7.4
AZT-3TC-TDF	172	1.2
d4T-3TC-EFV	33	0.2
d4T-3TC-NVP	2234	16.0
d4T-TDF-FTC	32	0.2
ddI-3TC-EFV	119	0.9
ddI-3TC-NVP	153	1.1
TDF-3TC-EFV	1673	12.0
TDF-3TC-NVP	2665	19.1
Total	13983	100.0

ABC, abacavir; AZT, zidovudine; 3TC, lamivudine; NVP, nevirapine; EFV, efavirenz; ddi, didanosine; TDF, tenofovir; FTC, entricitabine (often inter changeable with 3TC)

### Incidence of ADR and severity

During a median study duration of 3.5 years (IQR 1.4 –5.9) and 52,411 person-years (py) of observation, a total of 1485 incident ADRs were reported with an incidence rate (IR) of 28.3 (95% confidence interval [CI] 26.9:29.8) ADRs per 1000 person-years (py) of ART. The pattern of ADRs and severity are summarized in Table 3. The most commonly reported ADR was lipodystrophy (n=752) with an incident rate of 14.3 (95% CI 13.4:15.4) per 1000py, followed by anaemia (n=255) and peripheral neuropathy (n=108) with incident rates of 4.9 (95% CI 4.3:5.5) per 1000 py and 2.1 (95% CI 1.7:2.5) per 1000 py respectively. More than half (818 out of 1485; 55%) of the incident ADRs were grade three or four in severity with an IR of 15.6 (95% CI 14.6:16.8) per 1000 py. Of the grade 3 or 4 ADRs, Lipodystrophy had the highest incidence of 7.1(95% CI 6.4:7.9) per 1000 py. Most of the anaemia was grade three or four in severity, with an incident rate of 4.1(95% CI 3.6:4.7) per 1000 py. Other commonly reported grade 3 or 4 ADRs included peripheral neuropathy (n=38) and skin rash with itching (n=36).

**Table 3:** Incident rate of antiretroviral therapy-associated ADR according to the severity

ADR type according to Organ/system	Incident rate per 1000 py (95% CI) [number of events]		

	All severity	grade 1 and 2	Grade 3 & 4
All ADR	28.33 (26.92:29.81) [1485]	2.75 (1.81:3.75) [668]	15.61 (4.57:16.72) [818]
**Metabolic symptoms**			
Lipodystrophy	14.34 (13.36:15.41) [752]	7.25 (6.56:8.02) [380]	7.10 (6.41:7.86) [372]
Gynaecomastia	0.23 (0.13:0.4) [12]	0.17 (0.09:0.33) [9]	0.06 (0.02:0.18) [3]
**Systemic symptoms**			
Anaemia	4.87 (4.3:5.5) [255]	0.8 (0.59:1.08) [42]	4.06 (3.55:4.65) [213]
Hypersensitivity reaction	0.15 (0.08:0.31) [8]	0.07 (0.03:0.2) [4]	0.08 (0.03:0.2) [4]
Fever	0.04 (0.01:0.15) [2]	0.04 (0.01:0.15) [2]	0 (0:0) [0]
Headache	0.04 (0.01:0.15) [2]	0 (0:0) [0]	0.04 (0.01:0.15) [2]
**Skin and appendages**			
Rash and itching	1.68 (1.37:2.07) [88]	0.99 (0.76:1.3) [52]	0.69 (0.5:0.95) [36]
SJS	0.4 (0.26:0.61) [21]	0.17 (0.09:0.33) [9]	0.23 (0.13:0.4) [12]
Erythema multiforme	0.86 (0.64:1.15) [45]	0.17 (0.09:0.33) [9]	0.69 (0.5:0.95) [36]
Exfoliative skin lesions	0.1 (0.04:0.23) [5]	0.06 (0.02:0.18) [3]	0.04 (0.01:0.15) [2]
Hyperpigmentation (skin/nail)	0.25 (0.14:0.43) [13]	0.15 (0.08:0.31) [8]	0.1 (0.04:0.23) [5]
Photosensitivity reaction	0.02 (0.00269:0.14) [1]	0.02 (0.003:0.14) [1]	0 (0:0) [0]
**Peripheral nervous** **system**			
Peripheral neuropathy	2.06 (1.71:2.49) [108]	1.34 (10.6:1.69) [70]	0.73 (0.53:1) [38]
**Central nervous** **system**			
Nightmares	0.44 (0.29:0.66) [23]	0.44 (0.29:0.66) [23]	0 (0:0) [0]
Insomia	0.38 (0.25:0.59) [20]	0.38 (0.24:0.59) [20]	0 (0:0) [0]
Anxiety/restlessness/ loss of concentration	0.27 (0.16:0.45) [14]	0.06 (0:0.18) [3]	0.21 (0.12:0.38) [11]
Irrational talk/aggression	0.25 (0.14:0.43) [13]	0.11 (0.05:0.25) [6]	0.13 (0.06:0.28) [7]
Somnolence	0.15 (0.08:0.31) [8]	0.08 (0.03:0.2) [4]	0.08 (0.03:0.2) [4]
Hallucination	0.08 (0.03:0.2) [4]	0.02 (0.003:0.14) [1]	0.06 (0.02:0.18) [3]
Seizures	0.06 (0.02:0.18) [3]	0.02 (0.003:0.14) [1]	0.04 (0.01:0.15) [2]
**Gastro-intestinal**			
Nausea and vomiting	0.59 (0.42:0.84) [31]	0.02 (0.11:0.35) [10]	0.4 (0.26:0.61) [21]
Diarrhoea	0.48 (0.32:0.71) [25]	0.42 (0.28:0.64) [22]	0.06 (0.02:0.18) [3]
Abdominal pain	0.11 (0.05:0.25) [6]	0.08 (0.03:0.2) [4]	0.04 (0.01:0.15) [2]
**Hepatic symptoms**			
Jaundice	0.53 (0.37:0.77) [28]	0.29 (0.17:0.47) [15]	0.25 (0.14:0.43) [13]
**Renal symptoms**			
Oliguria/edema	0.25 (0.14:0.43) [13]	0.08 (0.03:0.2) [4]	0.17 (0.09:0.33) [9]
**Others**			
Muscle cramps	0.04 (0.01:0.15) [2]	0.04 (0.01:0.15) [2]	0 (0:0) [0]
Bone pain	0.04 (0.01:0.15) [2]	0 (0:0) [0]	0.02 (0:0.14) [1]
Deepening of voice	0.02 (0.002:0.14) [1]	0.02 (0.003:0.14) [1]	0 (0:0) [0]

### The onset of adverse drug reactions

The IR of ADRs was almost two times higher in the first year of ART compared to subsequent years; 42.51 (95% CI 39.0:46.3) per 1000 py for ≤1year of ART versus 24 (95% CI 22.5:25.5) per 1000 py for >1 year of ART, crude incidence rate ratio (IRR) 1.77 (95% CI 1.59:1.97) (Table 4). For specific ADRs, the incidence of metabolic symptoms, lipodystrophy, and gynecomastia, unlike other ADRs, were lower by about 82% and 35%, respectively, during the first year of therapy compared to subsequent years. Anemia had almost seven times higher incidence, while hypersensitivity reactions were higher by about 23 times in the first year compared to subsequent years. Adverse reactions involving skin and appendages such skin rash with itching, Stevenson Johnsons syndrome, and hyperpigmentation were higher by 21, 31, and 8 times respectively, during the first year of therapy compared to subsequent years. Similarly, Nervous system disorders such as peripheral neuropathy, nightmares, insomnia, anxiety/restlessness, and hallucination had about 5, 3, 8, 12, 3 times higher incidence respectively during the first year of therapy.

**Table 4:** Comparative Incidence of ADRs during the first and subsequent years of antiretroviral treatment at Jos University Teaching Hospital

ADR type according to Organ/system	Incident rate per 1000py (95% CI) [number of events]			Incident rate ratio (95% CI) a versus b

	Overall	Treatment period ≤1 year (a)	Treatment period >1 year (b)	
All events	28.33 (26.92–29.81) [1485]	42.51 (39.02:46.31) [524]	23.98 (22.51:25.54) [961]	1.77 (1.59:1.97)
**Metabolic** **symptoms**				
Lipodystrophy	14.34 (13.36–15.41) [752]	3.24 (2.38:4.42) [40]	17.76 (16.51:19.12) [712]	0.18 (0.13:0.25)
Gynaecomastia	0.23 (0.13–0.4) [12]	0.16 (0.04:0.65) [2]	0.25 (0.13:0.46) [10]	0.65 (0.07:3.05)
**Systemic** **symptoms**				
Anaemia	4.87 (4.3–5.5) [255]	13.79 (11.87:16.03) [170]	2.12 (1.71:2.62) [85]	6.5 (4.98:8.54)
**Hypersensitivity** **reaction**	0.15 (0.08–0.31) [8]	0.57 (0.27:1.19) [7]	0.02 (0:0.18) [1]	22.76 (2.92:1025.8)
Fever	0.04 (0.01–0.15) [2]	0.16 (0.04:0.65) [2]	0 (0:0) [0]	
Headache	0.04 (0.01–0.15) [2]	0.08 (0.01:0.58) [1]	0.02 (0:0.18) [1]	
**Skin and** **appendages**				
Rash and itching	1.68 (1.37–2.07) [88]	6.17 (4.92:7.72) [76]	0.3 (0.17:0.53) [12]	20.59 (11.13:41.6)
SJS	0.4 (0.26–0.61) [21]	1.54 (0.98:2.42) [19]	0.05 (0.01:0.2) [2]	30.89 (7.45:273.49)
Erythema multiforme	0.86 (0.64–1.15) [45]	3.24 (2.38:4.42) [40]	0.12 (0.05:0.3) [5]	26.01 (10.27:84.45)
Exfoliative skin lesions	0.1 (0.04–0.23) [5]	0.24 (0.08:0.75) [3]	0.05 (0.01:0.2) [2]	4.88 (0.56:58.39)
Hyperpigmentation (skin/nail)	0.25 (0.14–0.43) [13]	0.57 (0.27:1.19) [7]	0.15 (0.07:0.33) [6]	7.59 (1.73:45.47)
Photosensitivity	0.02 (0.00269–0.14) [1]	0.08 (0.01:0.58) [1]	0 (0:0) [0]	
**Peripheral** **nervous system**				
Peripheral neuropathy	2.06 (1.71–2.49) [108]	5.11 (3.99:6.54) [63]	1.12 (0.84:1.5) [45]	4.55 (3.06:6.83)
**Central** **nervous system**				
Nightmares	0.44 (0.29–0.66) [23]	0.89 (0.49:1.61) [11]	0.3 (0.17:0.53) [12]	2.98 (1.19:7.38)
Insomia	0.38 (0.25–0.59) [20]	1.14 (0.67:1.92) [14]	0.15 (0.07:0.33) [6]	7.59 (2.74:24.09)
Anxiety/ restlessness	0.27 (0.16–0.45) [14]	0.89 (0.49:1.61) [11]	0.07 (0.02:0.23) [3]	11.92 (3.15:66.55)
Irrational talk/aggression	0.25 (0.14–0.43) [13]	0.81 (0.44:1.51) [10]	0.07 (0.02:0.23) [3]	10.84 (2.79:61.29)
Somnolence	0.15 (0.08–0.31) [8]	0.41 (0.17:0.97) [5]	0.07 (0.02:0.23) [3]	5.42 (1.05:34.9)
Hallucination	0.08 (0.03–0.2) [4]	0.16 (0.04:0.65) [2]	0.05 (0.01:0.2) [2]	3.25 (0.24:44.86)
Seizures	0.06 (0.02–0.18) [3]	0.08 (0.01:0.58) [1]	0.05 (0.01:0.2) [2]	1.63 (0.03:31.23)
**GI symptoms**				
Nausea and vomiting	0.59 (0.42–0.84) [31]	1.95 (1.3:2.9) [24]	0.17 (0.08:0.37) [7]	11.15 (4.66:30.64)
Diarrhoea	0.48 (0.32–0.71) [25]	0.24 (0.08:0.75) [3]	0.55 (0.36:0.83) [22]	0.44 (0.08:1.48)
Abdominal pain	0.11 (0.05–0.25) [6]	0.16 (0.04:0.65) [2]	0.1 (0.04:0.27) [4]	1.63 (0.15:11.34)
**Hepatic** **symptoms**				
Jaundice	0.53 (0.37–0.77) [28]	1.46 (0.92:2.32) [18]	0.25 (0.13:0.46) [10]	5.85 (2.56:14.19)
**Renal** **symptoms**				
Oliguria/body swelling	0.25 (0.14–0.43) [13]	0.57 (0.27:1.19) [7]	0.15 (0.07:0.33) [6]	3.79 (1.09:13.66)
**Others**				
Muscle cramps	0.04 (0.01–0.15) [2]	0.04 (0.01–0.15) [2]		
Bone pain	0.04 (0.01–0.15) [2]		0.04 (0.01–0.15) [2]	
Deepening of voice	0.02 (0.002–0.14) [1]		0.02 (0.002–0.14) [1]	

### Predictors of ADR

Cox proportional hazard analysis (Table 5) revealed several baseline patient and regimen factors predictive of ADRs. The hazard of ADRs was 17% higher in females compared to males; adjusted hazard ratio (aHR) 1.17; 95% confidence interval (CI):1.03–1.33. However, when the hazard of ADRs was limited to grade 3 or 4 ADRs, gender was not a significant risk factor. There was a 2% increase in the hazard of all ADRs and grade 3 or 4 ADRs for every year increase in age. Patients with HIV/HBV co-infection at treatment initiation had a 24% lower hazard of ADRs compared to those who were HBV negative. Compared to those with pre-treatment WHO clinical stage 3 or 4 diseases, patients with WHO clinical stage 1 or 2 diseases had a 14% greater hazard of ADRs. However, when the ADRs were restricted to grade 3 or 4 ADRs, WHO clinical stage did not predict the risk of ADRs. Considering the ART regimen at treatment initiation, there was no significant difference between efavirenz and nevirapine in the hazard of ADR. For the nucleoside reverse transcriptase inhibitors, compared with tenofovir, the hazard of ADR was increased by 81%, 72%, and 93% in patients who initiated ART containing the abacavir, zidovudine, and didanosine respectively, while stavudine increased the hazard of ADRs 11-fold.

**Table 5:** Baseline patient and regimen characteristics and adjusted hazard of adverse drug reactions among patients on ART January 2004 - December 2015

Characteristics	Any ADR		Severe (grade 3 or 4) ADR	
	
	aHR (95% CI)	*P* value	aHR (95% CI)	*P*-value
Female	1.17 (1.03 – 1.33)	.014	1.12 (0.95 – 1.34)	0.188
Age, years	1.02 (1.02 – 1.03)	<0.001	1.02 (1.01 – 1.03)	<0.001
HBsAg positive	0.76 (0.35 – 0.89)	.001	0.79 (0.64 – 0.97)	0.028
TB positive	0.97 (0.84 – 1.13)	.701	1.01 (0.81 – 1.24)	0.986
WHO clinical stage 1 or 2	1.14 (1.01 – 1.30)	.042	0.89 (0.75 – 1.07)	0.228
CD4 count .100 cells/mm3	1.07 (0.95 – 1.21)	.276	0.90 (0.76 – 1.06)	0.214
Efavirenze versus nevirapine	0.83 (0.66 1.02)	.074	0.88 (0.66 – 1.16)	0.356
Abacavir*	1.81 (1.11 – 2.95)	.018	1.95 (0.99 – 3.84)	0.054
Zidovudine*	1.72 (1.39 – 2.14)	<0.001	2.54 (1.89 – 3.43)	<0.001
Stavudine*	10.39 (8.34 – 12.87)	<0.001	10.29 (7.61 – 13.92)	<0.001
Didanosine*	1.93 (1.17 – 3.18)	.010	2.71 (1.45 – 5.07)	0.002

ADR; adverse drug reaction, aHR; adjusted hazard ratio, HBsAg; hepatitis B serum antigen, TB; pulmonary tuberculosis, WHO; World Health Organization

Males compared with females, HBsAg positive patients compared with HBsAg negative patients, PTB positive patients compared with PTB negative, CD4 ≥100 cell/mm^3^ compared with CD4 cell count >100 cells/mm^3^, Efavirenz compared with nevirapine

* compared with tenofovir

## Discussion

This study found that, apart from metabolic syndromes such as lipodystrophy, the incidence of different types of adverse drug reactions associated with first-line ARVs were significantly higher in the first year of treatment compared to succeeding years of therapy. Generally, the Incidence of ADRs was almost double in the first year of therapy compared to subsequent years.

The finding of higher Incidence of ADRs during the first year of ART in our study is consistent with the finding of other studies. A Brazilian study found that most of the adverse reactions occurred before the 4^th^ month of treatment 21, while a report from Nigeria suggested that ADRs are more likely during the first six months of therapy 9. The higher incidence during the first year of ART might be the expression of a mechanism of intrinsic intolerance 22 as well as suggestive of a higher prevalence of early-onset ADRs. Also, auto-induction associated non-nucleoside reverse transcriptase inhibitors such nevirapine may result in an initial drug overexposure in the initial 2–4 weeks of therapy 23. The higher plasma concentrations during the first weeks of therapy because of auto-induction could, in part, explain the high Incidence of certain ADRs within the first year. These data highlight the need for closer monitoring of patients during the first year of ART, as it represents the period of greatest risk for most ADRs.

With an incident rate ratio of over 20, the risk of skin rash and other hypersensitivity reactions was greatest in the first years of therapy compared to subsequent years. Although we did not evaluate the risk factors for specific ADRs in this study, the early onset of skin rash and hypersensitivity reactions observed in the study is not unconnected with the use of nevirapine in 78% of the study participants (Table 2). The association of nevirapine with early onset of drug rash with eosinophilia and systemic symptoms (DRESS) has been documented in the literature 24–28. Other adverse drug reactions with a comparatively higher incidence during the first year of ART included anemia, central nervous system symptoms, and gastrointestinal disturbances. Our finding of a higher incidence of anaemia is in the first year of therapy is consistent with several other reports^29^–^31^. In agreement with our study findings, previous studies reported that central nervous system disorders, particularly those associated with the use of efavirenz, which was used in 21% of our study patients, are more common in the first year of ART 32–35. Neuropsychiatric ADRs are risk factors for poor medication adherence and treatment discontinuation. To mitigating the negative consequences of ART-related neuropsychiatric ADRs on treatment success, it is recommended that neuropsychiatric screening should form part of the routine care, particularly in the first year ART.

Unlike other ADRs observed in this study, morphological changes involving the redistribution of body fat (lipodystrophy) was delayed in onset with about 85% lower incidence during the first year of ART compared to subsequent years of treatment in a univariate analysis. This result agrees with others, which found that lipodystrophy was associated with the duration of ART^36^ with patients on ART for greater than two years at a greater risk of lipodystrophy^37^. Adverse drug reactions with delayed onset are particularly important as they may compromise the success of therapy in patients who are already stable on ART 5. Monitoring of patients for ADRs throughout antiretroviral therapy is, therefore, imperative to support early identification and treatment ADRs.

Some patients' and regimen characteristics increased the risk of ADRs to NNRT-based ART regimen in our study sample. A higher rate of ADRs was observed in females compared to males. In agreement with our study finding, an earlier review of gender differences in ADRs reported higher rates of ADRs in females on ART compared to males 38. The reason for the higher rates of ADRs in females compared to males merits further investigation in our study setting, particularly, that females account for the majority of patients on ART in this setting. Also, it is important to prioritize monitoring of females initiated on ART and provide timely interventions to mitigate the negative impact of drug toxicity such as poor adherence to treatment, treatment discontinuation, poor health outcomes, and reduction in the quality of life.

Consistent with the finding of other African studies 39,40, the risk of ART-related ADRs increased with advancing age. The finding of this study and that of similar studies highlights the challenge of drug-related morbidity among older patients initiating ART. Thus, strategies to improve the safety of ARV medicines should be carefully considered when initiating older patients on ART. Commencing ART at WHO clinical stage 1 or 2 disease was associated with a greater hazard of ADRs compared to treatment initiation at WHO clinical stage 3 or 4. There is the heterogeneity of findings regarding the association of disease stage at ART initiation with ADRs rates. Consistent with our study results, some evidence suggests that patients who initiate treatment at an early HIV disease stage are at higher risk of certain ADRs 41. For instance, a greater risk of NNRTI-related rash was reported in persons with earlier HIV disease after starting therapy 27. Another possible explanation for the higher incidence of ADRs in asymptomatic patients (WHO clinical stage 1 or) observed in this study is the fact that our study focused on clinical ADRs. Because of the focus on clinical ADRs, some clinical adverse drug events which overlapped with symptoms of HIV/AIDS in symptomatic patients (WHO stage 3 or 4) may have been missed, potentially resulting in under-reporting of ADRs in symptomatic patients. In contrast to our study finding, the HIV disease stage was not predictive of ADRs in a multicentre randomized controlled study 42. With the adoption of WHO test and treat strategy 43, ARV regimen should be carefully selected for asymptomatic patients initiating ART bearing in mind the possibility of greater risk of ADRs.

The association of the type of ARV regimen with the incidence of ADRs was studied, providing useful information for the management of antiretroviral therapy. Although the cumulative incidence of ADRs was higher in patients who initiated ART containing NVP compared to EFV (119 per 1000 compared to 58 per 1000), the multivariate Cox regression analysis did not reveal a difference in hazard of ADRs between the two drugs. Our study result is consistent with a previous study report that suggested that the short- and long-term toxicity and withdrawal rates of the two drugs were comparable 44. When the NRTIs were compared, the use of stavudine (11 times higher hazard) and didanosine (93% greater hazard) was associated with a significant risk of ADRs compared to tenofovir. However, stavudine and didanosine are no longer recommended for first-line therapy because of their greater potential for toxicity 45, and their use has been discontinued at the study site. In line with findings in the literature 46,47, the risk of ADRs was also significantly higher in patients exposed to AZT compared to TDF.

## Strengths and Limitations of the study

The retrospective cohort study design made it possible to examine a large longitudinal data of over 13,000 patients, spanning over a decade in a resource-limited setting. The electronic documentation with dates of occurrence of ADRs made it possible to describe the incidence rate and profile of ADRs in the studied population. Despite the strengths of the study, the following limitations should be considered in interpreting our results. 1) The study was conducted at one site; hence cautious generalization of the study results is advised, as individuals may exhibit variable responses to drugs. 2) The study may have under-reported the incidence of ADRs in the studied population as it focused more on clinical ADRs and did not analyze laboratory markers of certain ADRs. 3) Furthermore, we observed a trend toward documenting the first ADRs reported by patients. The report of subsequent ADRs was very sparse and could not be addressed due to the retrospective nature of the study.

## Conclusion

Although ART-related ADRs may occur at any time during therapy, the first year of ART is the period of greatest risk of ADRs. For NNRTI-based ART, the most significant ADRs during the first year of therapy include hypersensitivity reactions, anemia, and CNS disturbances. Strategies for early identification and management of ADR during this period are required for individual and programmatic treatment success in resource-limited settings.
